# A novel role of the TNF-α/p65/IRF1 axis in aggravating rheumatoid arthritis through the induction of fibroblast-like synoviocytes pyroptosis

**DOI:** 10.3389/fphar.2026.1896639

**Published:** 2026-07-31

**Authors:** Qian Jia, Xiuli Zhang, Li Jiang, Zhenchun Zhang, Zunzhong Li, Yuling Wang, Peng Yan

**Affiliations:** 1 Department of Rheumatology and Immunology, Linyi People’s Hospital Affiliated to Shandong Second Medical University, Linyi, China; 2 Department of Rheumatology and Immunology, Linyi People’s Hospital, Shandong Provincial Clinical Research Center for Immune Diseases and Gout, Linyi, China

**Keywords:** CASP1, fibroblast-like synoviocytes, GSDMD, IRF1, pyroptosis, rheumatoid arthritis

## Abstract

**Introduction:**

Rheumatoid arthritis (RA) is a chronic inflammatory disease characterized by aberrant proliferation and activation of fibroblast‐like synoviocytes (FLSs), leading to production of inflammatory mediators. Although interferon regulatory factor 1 (IRF1) is known to participate in immune regulation, its specific role and upstream activation mechanism in RA‐FLSs remain elusive.

**Methods:**

IRF1 expression was assessed in RA synovial tissues, collagen‐induced arthritis (CIA) mouse synovium, and TNF‐α‐stimulated RA‐FLSs. Functional assays evaluated pyroptosis after IRF1 knockdown. Mechanistic investigations included ChIP‐seq, Cut‐and‐Run, and luciferase reporter assays to identify IRF1 target genes and promoter binding. *In vivo*, CIA mice were treated with IRF1 modulation to evaluate arthritis severity.

**Results:**

IRF1 was significantly upregulated in RA tissues and TNF‐α‐treated FLSs. IRF1 suppression attenuated pyroptosis and reduced pyroptosis‐associated proteins (CASP1, GSDMD‐N, IL‐1β, IL‐18). TNF‐α activated p65, which directly bound to the IRF1 promoter, increasing IRF1 expression. IRF1 directly bound to the CASP1 promoter, enhancing CASP1 expression and pyroptosis. In CIA mice, IRF1 modulation markedly alleviated arthritis symptoms.

**Conclusion:**

The TNF‐α–p65–IRF1–CASP1 axis drives pyroptosis in RA‐FLSs and contributes to RA pathogenesis, identifying IRF1 as a promising therapeutic target value.

## Graphical Abstract

**Figure:**
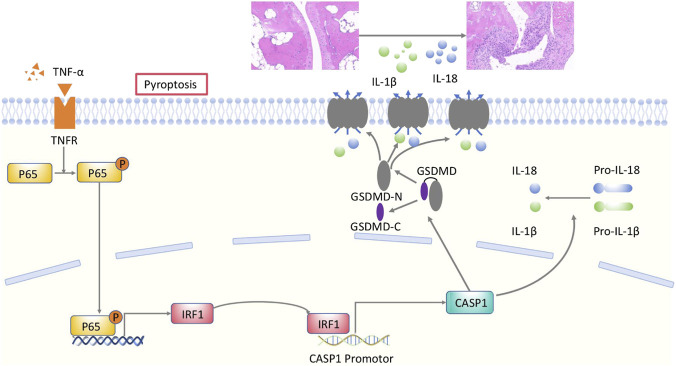

## 1 Introduction

Rheumatoid arthritis (RA) is a chronic autoimmune disease. It stands as one of the most widespread immune-mediated conditions globally, primarily manifesting as inflammatory arthritis defined by symmetrical, polyarticular pain and swelling. It predominantly causes synovial inflammation and adjacent cartilage and bone erosion, ultimately causing disability in patients (Konzett and Aletaha, 2024). A hallmark pathological feature of RA is the uncontrolled proliferation of fibroblast-like synoviocytes (FLSs), which produce large quantities of inflammatory mediators and matrix metalloproteinases.Thereby leading to the degradation of surrounding cartilage and bone tissue, ultimately resulting in disability for patients (Nemeth et al., 2022). The primary therapeutic agents for rheumatoid arthritis comprise disease-modifying anti-rheumatic drugs (DMARDs), non-steroidal anti-inflammatory drugs, glucocorticoids, and biologics. These medications alleviate synovitis and mitigate systemic inflammation (Deane et al., 2025). Whilst effective in controlling RA symptoms, they fail to fundamentally halt disease progression. Therefore, there is an urgent need to investigate the mechanisms underlying RA and to discover novel therapeutic strategies. As a result, there is a pressing need to explore RA pathogenesis and to pursue novel therapeutic strategies (Sebbag et al., 2025).

In RA, FLSs are activated by TNF-α secreted from macrophages and Th1 cells within the synovial microenvironment, thereby undergoing excessive proliferation. This leads to increased production of inflammatory cytokines and overexpression of matrix metalloproteinases, resulting in collagen degradation, bone destruction, and subsequent joint erosion (Lee et al., 2013). The TNF/TNFR1-NF-κB signalling pathway further contributes to inflammation through both direct transcriptional regulation of inflammatory genes and indirect promotion of cell death (van Loo and Bertrand, 2023).

Fibroblasts in chronic inflammatory diseases, including rheumatoid arthritis, actively recruit and maintain inflammatory leukocytes, thereby emerging as the principal producers of pro-inflammatory cytokines and catalysts of tissue damage (Zou et al., 2025). FLSs constitute a pivotal cell type within the synovial lining and sublining layers. They are responsible not only for extracellular matrix production but also for regulating the intra-articular microenvironment by releasing matrix metalloproteinases (Smolen et al., 2018). Their normal function is critical for maintaining environmental homeostasis (Meng et al., 2024; Triaille et al., 2025). In RA, FLSs exhibit semi-autonomous characteristics, including anchorage-independent growth, loss of contact inhibition, and increased expression of disease-associated cytokines, chemokines, matrix metalloproteinases and their tissue inhibitors (Meng et al., 2024; Smith et al., 2023). In TNF-α-induced RA-FLSs, Gal-9 knockdown was found to suppress proliferation, migration, invasion, and the expression of cytokines and MMPs, while concurrently promoting apoptosis, through mechanisms involving the MAFB and PI3K/AKT pathway (Jia et al., 2024), Current research indicates that multiple proteins, drugs, and cytokines can modulate FLS proliferation, apoptosis, migration, invasion, and inflammatory responses. Consequently, FLSs research has become a focal point within the RA field (Nemeth et al., 2022; Kugler et al., 2023).

Over the past several years, increasing attention has been directed toward the involvement of pyroptosis—a form of programmed cell death—in the pathogenesis of rheumatoid arthritis (Gu et al., 2022; Zhai et al., 2022). This form of cell death is defined by swift membrane disruption, along with the secretion of pro-inflammatory cytokines, most notably IL-1β and IL-18 (Demarco et al., 2022). Upon recognition of exogenous pathogenic molecules, such as lipopolysaccharide (LPS), macrophages rapidly activate an inflammatory cascade. This response facilitates the formation of the NLRP3 inflammasome—a multiprotein complex consisting of the NOD-like receptor family pyrin domain-containing protein 3 (NLRP3), the adaptor protein ASC(apoptosis-associated speck-like protein containing a CARD, ASC), and the precursor enzyme pro-caspase-1. Through pro-caspase-1-mediated proteolytic cleavage, the cytokines IL-1β and IL-18 are subsequently released. Pyroptosis of macrophages not only intensifies inflammatory reactions but also stimulates pathological proliferation of synovial fibroblasts and enhances the production of matrix metalloproteinases. These effects contribute to aggravated tissue injury and can eventually lead to irreversible bone erosion in rheumatoid arthritis (Burdette et al., 2021; Lawlor et al., 2024; Bai et al., 2025; Zhang et al., 2024). In addition to pyroptosis, other novel forms of programmed cell death have recently been implicated in RA pathogenesis. Cuproptosis, a copper-dependent cell death pathway, has emerged as a contributor to RA progression. Lin et al., through integrated bioinformatics analysis and single-cell RNA sequencing, identified ten differentially expressed cuproptosis-related genes in RA and revealed their associations with immune infiltration and diagnostic efficacy, providing novel insights into the molecular mechanisms and potential therapeutic targets in RA (Li et al., 2025). Similarly, ferroptosis—an iron-dependent form of non-apoptotic cell death—has also been linked to RA. Li et al. identified 34 potential differentially expressed ferroptosis-related genes in RA through bioinformatics analysis, among which GABARPL1, DUSP1, JUN, and MAPK8 were validated as diagnostic biomarkers and therapeutic targets, primarily enriched in the HIF-1, FoxO, and Ferroptosis signaling pathways (Li et al., 2023). These findings collectively underscore the multifaceted roles of diverse programmed cell death modalities—including pyroptosis, cuproptosis, and ferroptosis—in the complex pathophysiology of RA. However, pyroptosis in FLS remains poorly studied. To date, whether TNF-α alone directly activates the classical CASP1/GSDMD-dependent pyroptotic cascade in RA-FLSs remains incompletely understood, and direct mechanistic evidence is still limited. Earlier studies have demonstrated that co-stimulation with TNF-α and CRT induces caspase-1 p20 expression and promotes IL-1β secretion in RA-FLSs (Liu et al., 2019). More recently, it was reported that TNF-α alone significantly enhances the activation of caspase-3 and GSDME, thereby inducing GSDME-mediated pyroptosis in RA monocytes (Zhai et al., 2022). Other investigators have found that TNF-α activates the NLRP3 inflammasome in RA-FLSs, thereby promoting pyroptosis (Liu et al., 2024). In addition, TNF-α stimulation of the human synovial fibroblast cell line MH7A has been shown to increase lactate dehydrogenase (LDH) release in the culture supernatant, elevate pro-inflammatory cytokine levels and intracellular caspase-1 activation, and upregulate the expression of NLRP3/GSDMD pathway-related proteins (Zhang et al., 2026). Collectively, these findings indicate that TNF-α promotes the pyroptotic process in RA-FLSs. Studies from our research group have yielded similar conclusions, demonstrating that TNF-α is highly expressed in RA-FLSs and plays a critical role in pyroptosis through activation of nuclear factor-κB (NF-κB), promoting the expression of NLRP3, CASP1, GSDMD-N, and IL-1β in RA-FLSs (Ren et al., 2023). Furthermore, TNF-α-stimulated RA-FLSs exhibit upregulated expression of pyroptosis-related proteins, including GSDMD, IL-1β, and NLRP3, along with significantly higher concentrations of IL-6, IL-1β, and IL-18 in the culture supernatant compared to the unstimulated group (Chen et al., 2023). Nevertheless, the current data are still insufficient to fully establish that TNF-α alone can activate the classical CASP1/GSDMD-dependent pyroptosis pathway, and direct mechanistic studies targeting this pathway remain limited.

Interferon Regulatory Factor 1 (IRF1) belongs to the interferon regulatory factor family of transcription factors. As the first transcription factor discovered in humans to bind the interferon-stimulated response element (ISRE) in target genes, IRF1 exerts its function through this specific interaction. Like all members of the IRF family, the N-terminal region of IRF-1 contains a DNA-binding domain comprising five tryptophan residues (Alsamman and El-Masry, 2018). IRF1 serves a pivotal function in interferon-mediated cellular injury, primarily by mediating apoptosis, promoting autophagy, and enhancing immune surveillance. IRF1 exerts significant influence in the regulation of both innate and adaptive immunity (Borden et al., 2007). Recent investigations highlight the significant involvement of IRF1 in a spectrum of pathologies, including tumor immunity, liver fibrosis, traumatic brain injury, degenerative diseases, and inflammatory disorders (Pang et al., 2025; Jang et al., 2025; Zhang et al., 2025a; Cui et al., 2025; Xin et al., 2025; Zhang et al., 2024b; Geng et al., 2024a; Deng et al., 2024).

IRF1 participates in the pathogenesis of multiple diseases by regulating pyroptosis. The IRF1/GBP5 (guanylate binding protein 5, GBP5) axis promotes cellular pyroptosis during osteoarthritis progression (Tang et al., 2024). IRF1 regulates pyroptosis in vascular endothelial cells to inhibit atherosclerosis (Fan et al., 2023). IRF1 contributes to pyroptosis in trophoblast cells, inducing miscarriage (Wang et al., 2023).

In rheumatoid arthritis, IRF1 similarly drives multiple inflammatory responses. Inhibiting IRF1-STAT1-driven chemokine expression reduces the severity of arthritis (Karonitsch et al., 2024), whilst TNF-α promotes inflammatory osteoclast formation via the IRF1/IFNβ/IFN axis (Xia et al., 2022). Nevertheless, whether IRF1 is involved in pyroptosis within RA-FLSs remains to be investigated. In the present study, we observed for the first time that IRF1 is markedly upregulated in RA synovium, arthritic joints of CIA mice, and TNF-α-induced RA-FLSs. Subsequently, Our findings establish IRF1 as a critical mediator of pyroptosis in RA-FLSs and a central driver of joint inflammatory responses. The exploration of its molecular mechanism ultimately revealed the pathogenic significance of IRF1 in RA, suggesting that targeting IRF1 could unlock new possibilities for therapeutic intervention.

## 2 Materials and methods

### 2.1 Clinical samples

Synovial tissue samples were collected from patients who underwent knee arthroplasty at Qilu Hospital of Shandong University and Linyi People’s Hospital between April and November 2022. The cohort consisted of eight patients with rheumatoid arthritis (RA) and five with osteoarthritis (OA), with groups balanced for gender, age, and disease activity. This study was approved by the institutional review boards of both hospitals (KYLL‐202111‐165‐1; Approval No. YX200237).

### 2.2 Mouse studies

All animal procedures were conducted in accordance with protocols approved by the Animal Care and Use Committee of Shandong University (Approval No.: 23,025). Eight-week-old male DBA/1 mice, obtained from Vitas River Laboratory Animal Technology Co., Ltd. (Beijing, China), were housed under standardized specific pathogen-free conditions: temperature 18 °1C–22 °C, relative humidity 50%–60%, noise ≤60 dB, lighting cycle 10–14 h, ammonia concentration ≤20 ppm, air exchange 8–20 times/h, and airflow 10–25 cm/min. Bedding was replaced weekly. The short hairpin RNA sequence targeting mouse IRF1 (shIRF1: TGG​CAT​ATG​CAG​ATG​GAC​A) was cloned into the pAV-U6-shRNA-CMV-Intron-GFP vector. Adeno-associated virus serotype 8 carrying siIRF1 (AAV8-siIRF1) was delivered into the hind paw via intra-articular injection 2 weeks prior to the induction of collagen-induced arthritis (CIA). The CIA model was established as previously reported (Wang et al., 2016; Vande Walle et al., 2014; Durie et al., 1993). Briefly, bovine type II collagen (Chondrex, Seattle, WA, United States of America) was emulsified with an equal volume of complete Freund’s adjuvant (Millipore Sigma, Burlington, MA, United States of America) on ice to form a stable emulsion. Eight-week-old male DBA/1 mice were randomly assigned to three groups (n = 4 per group): control, CIA, and CIA + AAV8-siIRF1. On day 0, mice in the CIA and CIA + AAV8-siIRF1 groups received a caudal injection of 100 µL of the emulsion. The control group underwent the same procedure concurrently with an equal volume of PBS (phosphate-buffered saline). A second vector injection, aimed at augmenting the immune response, was performed on day 21. Arthritis severity in each paw was evaluated using a standard 0–4 scoring system based on the extent of swelling and erythema. The scoring criteria were as follows: 0, no visible signs of inflammation; 1, mild erythema and swelling restricted to the ankle or mid-foot; 2, moderate erythema and swelling affecting the ankle or wrist; 3, severe erythema and edema involving the entire paw, including the digits; and 4, maximal inflammation with multi-joint involvement. The cumulative score for each animal was calculated as the sum of all four paw scores, with a maximum possible total of 16. On day 42, all mice were euthanized, and tissue specimens were harvested for subsequent analysis.

### 2.3 Single-cell sequencing data integration, processing and cell type annotation

In this study, we analysed single-cell RNA sequencing data from RA synovial samples within the GEO dataset GSE299518 (https://www.ncbi.nlm.nih.gov/geo/query/acc.cgi?acc=GSE299518). Initially, data preprocessing was performed using screening criteria (nFeature_RNA >200, nCount_RNA >200, percent.mito <20) to remove low-quality cells. Subsequently, the FindVariableFeatures function within the Seurat (v4.4.0) package was employed to select 2000 highly variable genes, and ScaleData was utilised for data normalisation. For dimensionality reduction, Principal Component Analysis (PCA) was employed for preliminary reduction. The optimal number of principal components (npcs) was selected based on the elbow rule as implemented in the ElbowPlot function, combined with cumulative variance coverage (retaining ≥80% cumulative variance) to select suitable principal components for subsequent analysis. Following principal component selection, the RunUMAP function was applied to perform UMAP dimensionality reduction based on PCA results, enabling visualisation of cell distribution.

Cell type annotation was performed according to the source literature (PMID: 40721606). Specific cell types are as follows: Fibroblasts (Fibroblast) 3,081, comprising CD142+ Fibroblasts (CD142+ Fibroblast) 310, DKK3+ Fibroblasts (DKK3+ Fibroblast) 409, and PRG4+ Fibroblasts (PRG4+ Fibroblast) 2,362. Other cell types comprised 1,047 macrophages, 49 monocytes, and 225 T cells. Subsequently,For the purpose of detecting differentially expressed genes in each cell type, the FindAllMarkers function from the Seurat (v4.4.0) package was applied. Finally, we used the DotPlot function from the scCustomize (v1.2.0) package to generate a bubble plot of highly variable genes, illustrating differences in gene expression across distinct cell types.

### 2.4 Differential expression analysis of IRF1 across cell types in synovium

To investigate the expression pattern of IRF1 across various cell types and fibroblast subsets, we first performed imputation on the single-cell RNA sequencing expression matrix. This preprocessing step, carried out using the Rmagic package (v 2.0.3), was aimed at reducing data sparsity prior to differential expression analysis. Subsequently, box plots were generated using the ggplot2 (v 3.4.4) and ggpubr (v 0.6.0) packages, with pairwise comparisons between groups conducted using the Wilcoxon signed-rank test. Secondly, IRF1 gene expression differences between fibroblasts and other cell types were assessed across all available samples. The original sample sizes for each population (fibroblasts: 3,081; macrophages: 1,047; T cells: 225; monocytes: 49) were utilised to preserve statistical power and accurately estimate the relative proportions of distinct cell populations present in the tissue. Here, unbalanced data were employed for overall comparisons between fibroblasts and other cell types. This approach reflects the biological reality that fibroblasts constitute the predominant cell population in synovial tissue and form the core IRF1-expressing group, where their abundance advantage inherently signifies biological significance while ensuring statistical test power. This combined advantage of cell abundance and expression distribution could be diluted by rebalanced sampling; thus, unbalanced data were prioritised in global comparisons to highlight their biological dominance. To further analyse fibroblast internal heterogeneity, given the significant sample size imbalance among its three subpopulations (PRG4+ Fibroblast: 2,362, DKK3+ Fibroblast: 409, CD142+ Fibroblast: 310), measures were taken to prevent the statistically dominant high-abundance subpopulations from inducing false positives or biases in statistical tests. Therefore, using the dplyr package (v 1.1.4), cell populations representing each fibroblast subtype were selected. A random subsampling method was employed to balance the number of cells across subtypes to the minimum sample size (310 cells), ensuring fairness in intergroup comparisons. A random seed (set. seed = 123) was set to guarantee reproducibility of results. Furthermore, sensitivity analysis was conducted through multiple random subsampling runs (n = 4, set. seed = 123) to validate the consistency of IRF1 expression trends across fibroblast subpopulations, thereby ensuring robustness of the findings.

### 2.5 Analysis of differences in conventional transcriptome data between RA and healthy patients, with a volcano plot and a box plot of differences

A total of 16 synovial biopsies from patients with advanced rheumatoid arthritis and seven synovial biopsies from healthy individuals (without joint disease) were sourced from the GEO database for transcriptomic analysis (GSE number GSE77298, https://www.ncbi.nlm.nih.gov/geo/query/acc.cgi?acc=GSE77298). The GEO2R tool was employed to standardise the GSE77298 data (using the RMA algorithm) and perform differential expression analysis (using the limma algorithm). Significantly differentially expressed genes were identified using the threshold |log2FC| > 1 and P < 0.01. Subsequently, ggplot2 (v 3.4.4) was used to generate a volcano plot showing the distribution of differentially expressed genes (significantly upregulated genes in red, significantly downregulated genes in blue, and others in grey). Download the IRF1 expression data from GSE77298 and utilise GraphPad Prism 10 to generate a box plot for differential expression analysis.

### 2.6 Immunohistochemistry (IHC)

IHC staining was performed in accordance with established procedures (Pinheiro et al., 2008) For antigen detection, the following primary antibodies were utilized: anti-IRF1 (1:100; CST, #8478, United States of America) and anti-CASP1 (1:100; Affinity, #AF5418, China), both of which recognize human and mouse tissues; anti-GSDMD-N antibody for human tissue (1:500; CST, #8478, United States of America); and anti-GSDMD-N antibody for mouse tissue (1:50; Affinity, #DF13758, China).

### 2.7 Immunofluorescence (IF)

Immunofluorescence staining was performed using a standardized protocol (Ge et al., 2022).

### 2.8 Cell isolation and culture

Rheumatoid arthritis fibroblast-like synoviocytes (RA-FLSs; catalog number CTCC-001-0385) were obtained from Zhejiang Missen Cell Technology Co., Ltd. and cultured at 37 °C in a humidified incubator with 5% CO_2_, using DMEM supplemented with 10% fetal calf serum, 100 IU/mL penicillin, and 100 μg/mL streptomycin. The medium was replaced every 48 h, and cells from passages three to eight were used for all experiments.

### 2.9 RNA extraction and real-time qPCR

We extracted total RNA from rheumatoid arthritis fibroblast-like synoviocytes (RA-FLSs) employing the RNA Fast 200 kit (Fastagen, Shanghai, China). The extracted RNA was then reverse transcribed into cDNA using a reverse transcription kit (AG, Accurate Biotechnology). For qRT-PCR analysis, cDNA was amplified with the SYBR Green PCR kit (AG, Accurate Biotechnology) on a CFX96 Touch™ real-time PCR detection system (Bio-Rad, Hercules, CA, United States of America), following the manufacturer’s protocol. The primer sequences utilized for the target genes are provided below. Homo-β-Actin Forward: GAA​GAG​CTA​CGA​GCT​GCC​TGA; Reverse: CAG​ACA​GCA​CTG​TGT​TGG​CG; Homo-IRF1 Forward: GAG​GAG​GTG​AAA​GAC​CAG​AGC​A; Reverse: TAG​CAT​CTC​GGC​TGG​ACT​TCG​A; Homo-CASP1 Forward: GTG​CAG​GAC​AAC​CCA​GCT​AT; Reverse: TGC​GGC​TTG​ACT​TGT​CCA​TT; Homo-p65 Forward: GAC​GCA​TTG​CTG​TGC​CTT​C; Reverse: TTG​ATG​GTG​CTC​AGG​GAT​GAC.

### 2.10 Western blot analysis (WB)

Following cell lysis and centrifugation, protein concentrations in the supernatants were determined using a BCA Protein Assay Kit. After adjusting to the desired concentration, samples were separated by electrophoresis on an appropriate polyacrylamide gel at a constant voltage of 150 V and subsequently transferred onto a PVDF membrane (Immobilon-P; Millipore). The membrane was then blocked with 5% skim milk on an orbital shaker for 1 h at room temperature, followed by overnight incubation with primary antibody at 4 °C. After washing with Tris-buffered saline containing 0.1% Tween-20, the membrane was incubated with an appropriate secondary antibody for 1 h at room temperature. Protein bands were visualized using an enhanced chemiluminescence (ECL) kit (Millipore). The following primary antibodies were used: anti-β actin (1:1,000; ZSGB-BIO, TA-09, China); anti-GAPDH (1:1,000; CST#5174; United States of America); anti-Histone H3 (1:1,000; Santa Cruz Biotechnology, #sc-517576; Dallas, TX) anti-IRF1 antibody (1:100; CST, #8478, United States of America); anti-CASP1 antibody (1:1,000; CST, #3866, United States of America); anti-cleaved-GSDMD antibody (1:1,000; CST, #36425, United States of America); anti-pp65 antibody (1:1,000; CST, #8242, United States of America); anti-p-pp65 antibody (Ser536) (1:1,000; CST, #3033, United States of America).

### 2.11 Enzyme-linked immunosorbent assay (ELISA)

To measure the levels of interleukin-1β (IL-1β) and interleukin-18 (IL-18), cell culture supernatants were collected, centrifuged at 2000 rpm for 5 min, and analyzed using ELISA kits obtained from Multiscience (Lianke) Biotechnology Co., Ltd. (Hangzhou, China), strictly following the manufacturer’s protocols.

### 2.12 Haematoxylin and eosin (HE)

For histopathological analysis, paw tissues were decalcified in 10% EDTA until fully demineralized, processed for paraffin embedding, and cut into 5 μm sections prior to hematoxylin and eosin (HE) staining. Histology scores were assessed as previously described (Wu et al., 2025; Orr et al., 2017).

### 2.13 Transfection of siRNA

We seeded RA-FLSs 24 h before transfection. Transfection of siRNA (Tsingke, Beijing, China) was carried out with jetPRIME® (Polyplus-transfection S.A., Illkirch, France). After 6–8 h of transfection, the culture medium was replaced, and cells were incubated for 24 h in the presence or absence of TNF-α (25 ng/mL, PeproTech, Rocky Hill, NJ, United States of America). Sequence of siRNA are as follows: si-IRF1#1:TCACCAAGAACCAGAGAAA; si-IRF1#2:GGUGAAUUUCAU GAGCUAA; si-pp65#1:CCCACGAGCTTCTAGGAAA; si-p65#2:GATTGAGGAGAAACGTAAA.

### 2.14 RNA sequencing (RNA-seq)

We harvested TNF-α-stimulated RA-FLSs after transfection with either siIRF1 or siNC and immediately lysed the cells in Trizol reagent for subsequent RNA extraction. Total RNA was purified with the RNA Prep Pure Plant Plus Kit (DP441, TIANGEN); all preparations exhibited excellent integrity (RIN 8–10). Libraries were constructed from the purified RNA, pooled, and sequenced on an Illumina NovaSeq platform using 150-bp paired-end reads. Following quantification of gene expression, differential expression analysis was performed with the edgeR package, applying significance cutoffs of adjusted p-value ≤0.05 and absolute log_2_ fold change ≥1.

### 2.15 Cleavage under targets and tagmentation (CUT&Tag)

Following the manufacturer’s instructions, the CUT&Tag assay was performed with the Hieff NGS® G-Type *In-Situ* DNA Binding Profiling Library Prep Kit for Illumina® (Yeasen). The promoter region of IRF1 spanning from −2000 to +100 was analyzed using the JASPAR CORE database (http://jaspar.genereg.net). The primer sequences employed for amplification of this region are provided below. IRF1 Site 1, Forward GCTTCTCTGAACCCCTT; Reverse AGT​AGT​TTT​CAA​GGT​CGC​CGT; IRF1 Site 2, Forward GAAAAACAGAGGTCCTG; Reverse AAG​AAG​GCA​GAA​AGC​CTT​CCC. The primary antibody is anti-p65 antibody (1:50; CST,#8242,United States of America).

### 2.16 Chromatin immunoprecipitation sequencing (ChIP-seq) analysis

TNF-α-treated RA-FLSs, cultured in DMEM with 10% FBS, were crosslinked with 1% formaldehyde for 10 min at room temperature, and the reaction was halted by adding 125 mM glycine for 5 min After washing with cold PBS, cells were lysed and nuclei were isolated. Chromatin was sheared to 200–500 bp fragments using a Covaris S220 ultrasonicator (175 W peak power, 10% duty factor, 200 cycles/burst, 5 min), with fragmentation efficiency verified by agarose gel electrophoresis. Sheared chromatin was incubated overnight at 4 °C with 5 µg of anti-IRF1 antibody (CST, #8478, United States of America) or normal rabbit IgG (CST, #2729) as a negative control. Immune complexes were captured with Protein A/G magnetic beads (Thermo Fisher Scientific, #26162) for 2 h at 4 °C, washed sequentially with low-salt, high-salt, LiCl, and TE buffers, and reverse-crosslinked at 65 °C overnight alongside reserved Input DNA. After RNase A and Proteinase K treatment, DNA was purified using the QIAquick PCR Purification Kit (QIAGEN, #28104). Sequencing libraries were prepared with the NEBNext Ultra II DNA Library Prep Kit for Illumina (NEB, #E7645) per manufacturer’s instructions and sequenced on an Illumina NovaSeq 6,000 platform (150 bp paired-end). Raw reads were quality-checked with FastQC (v0.11.9) and aligned to GRCh38/hg38 using Bowtie2 (v2.4.4). Peaks were identified with MACS2 (v2.2.7.1) at a q-value <0.05, and motif enrichment analysis within peak regions was performed with HOMER (v4.11).

### 2.17 Cleavage under targets and release using nuclease (Cut&Run) assays

For the CUT&RUN assay, we employed the Hyperactive pG-MNase CUT&RUN Assay Kit for PCR/qPCR (HD101, Vazyme) following the manufacturer’s instructions. The −2000 to +100 region of the CASP1 promoter was analyzed using the JASPAR CORE database (http://jaspar.genereg.net). Primer sequences used to target the CAPS1 promoter region are listed below: Forward GTC​CCT​TCC​ACC​TAT​GAG​CCT; Reverse GAC​TCG​CAT​GGG​GAA​TGT​AGC. The primary antibody is anti-IRF1 antibody (1:50; CST,#8478,United States of America).

### 2.18 Transmission electron microscope

To examine morphological changes associated with pyroptosis, cells subjected to TNF-α stimulation and IRF1 knockdown were processed for transmission electron microscopy (TEM). Following three washes with PBS, the samples were fixed in glutaraldehyde and subsequently post-fixed in 1% osmium tetroxide. Subsequent processing included dehydration through a graded isopropanol series, followed by embedding in a mixture of epoxy resin and 2% uranyl acetate. Ultrastructural imaging was ultimately performed using a Hitachi HT7700 microscope.

### 2.19 Luciferase reporter assay

To examine whether p65 regulates transcription via the IRF1 promoter, HEK293T cells were seeded into 24-well plates and cultured until they reached approximately 70% confluence. Subsequently, the cells were co-transfected with a p65-expressing plasmid, the pRL-TK internal control vector, and a pGL3-basic construct harboring the IRF1 promoter (synthesized by GenePharma, Shanghai, China); transfection was performed using jetPRIME reagent. At 48 h post-transfection, cells were harvested and lysed, and luciferase activity was measured using a dual-luciferase reporter assay kit (Yeast Biotechnology, Shanghai) on a Tecan Infinite E Plex system (Tecan, Switzerland).

### 2.20 DNA agarose gel electrophoresis experiment

Pour 100 mL of 1× TAE electrophoresis buffer into a pre-prepared clean flask. Add 2 g of agarose powder and shake thoroughly. Place the flask in the microwave, remove it every 30 s to shake, and continue shaking until boiling is reached, then remove the flask. Allow the flask to cool to room temperature, then add 10 µL of nucleic acid dye and gently swirl to ensure thorough mixing. Insert the comb into the gel plate, place it flat in a 4 °C refrigerator, and slowly pour the prepared solution into the plate. After a period, gently remove the comb. Place the gel into the electrophoresis tank and pour in 1× TAE buffer, ensuring the electrophoresis solution covers the gel by 3–5 mm. Lay a clean sealing membrane flat on the work surface. Select the samples, use a pipette to draw 10 µL of each DNA product, and add it to the gel well, submerged in the electrophoresis buffer. Add 10 µL of DNA marker to the central well to detect molecular weight and record the loading order. Connect the power supply, set the voltage to 65 V for 30 min, and press the start button. When the indicator reaches the designated position, switch off the power. Place the gel on the gel imaging system to analyse band sizes and product specificity relative to the DNA marker, and record the data.

### 2.21 Statistical analysis

We conducted all experiments at least three times independently. Data analysis and graphical representation were carried out with GraphPad Prism 10, and the data are reported as mean ± SEM. To determine statistical significance, Student’s t-test was used for two-group comparisons; for three or more groups, data were first checked for normality and homogeneity of variances. One-way ANOVA was applied when assumptions were met, with appropriate post hoc tests (Dunnett’s for comparisons against a common control, or Tukey’s for all pairwise comparisons); otherwise, Kruskal–Wallis test was used.

## 3 Results

### 3.1 IRF1 expression is upregulated in rheumatoid arthritis

**FIGURE 1:**
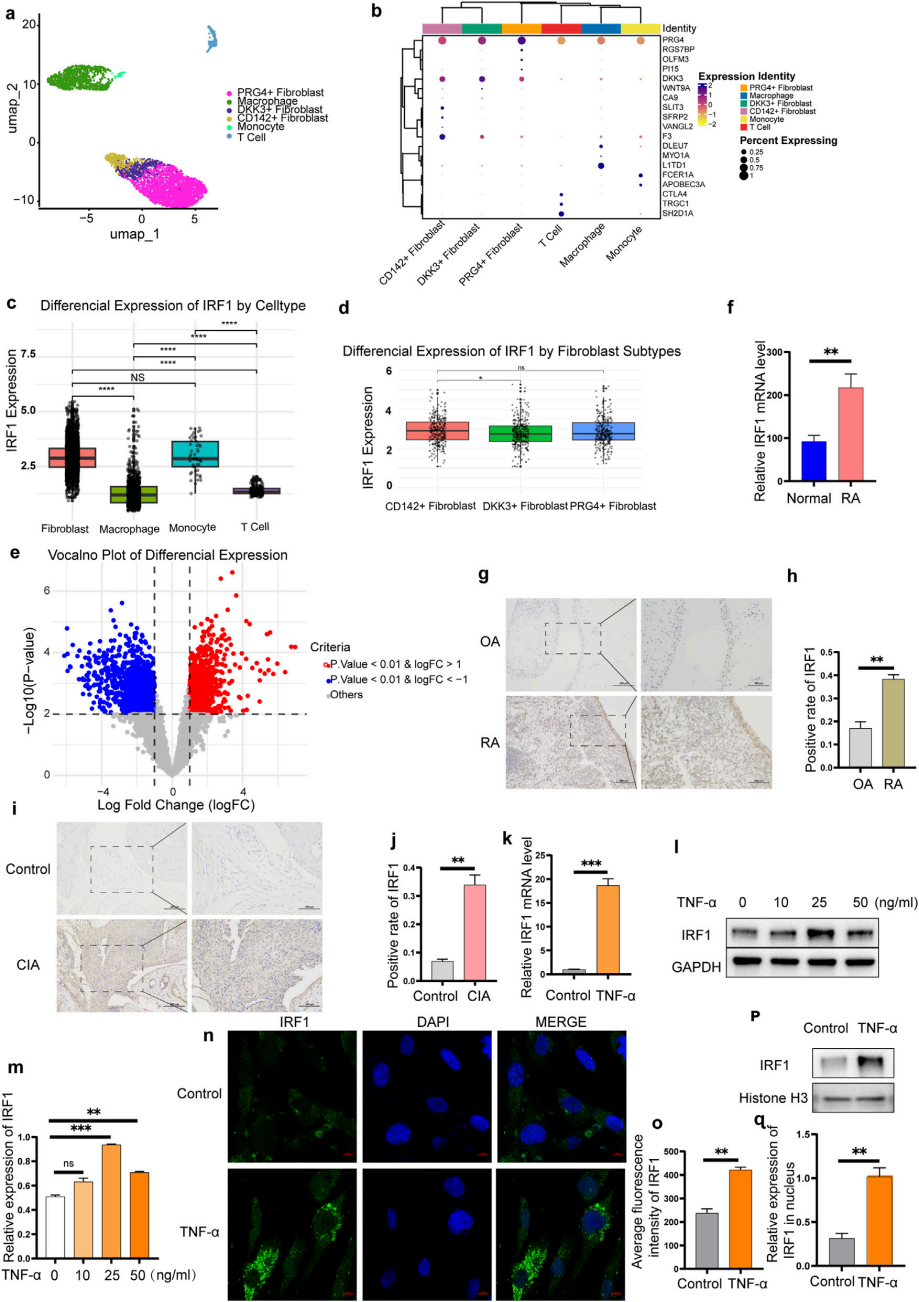
IRF1 is upregulated in synovial tissues of rheumatoid arthritis and TNF-α-treated FLSs. **(a)** Annotated RA single-cell UMAP visualisation (CD142+ Fibroblast, DKK3+ Fibroblast, PRG4+ Fibroblast, Macrophage, Monocyte, T cell).**(b)** Highly variable gene expression bubble heatmap. **(c)** Box plot of IRF1 expression differences between fibroblasts and other cell types. **(d)** Box plot of IRF1 expression differences across fibroblast subtypes. **(e)** Volcano plot of differentially expressed genes between RA and healthy controls in GSE77298 data. **(f)** Box plot of IRF1 expression differences in GSE77298 data between RA and healthy controls. **(g)** Representative images of IRF1 staining by immunohistochemistry in synovial tissue of Rheumatoid arthritis (RA, n = 8) and Osteoarthritis (OA, n = 5). Bar = 200 μm (left) or 100 μm (right). **(h)** Analysis of IRF1immunohistochemistry staining of (g). **(i)** Representative images of IRF1 staining by immunohistochemistry in synovial tissue of untreated mice (n = 4) and CIA model mice (n = 4). Bar = 100 μm (right) or 200 μm (left). **(j)** Analysis of IRF1immunohistochemistry staining of (i). **(k)** RT-qPCR analysis of IRF1mRNA in TNF-treated FLSs. **(l,m)** Western blot analysis of IRF1 protein expression stimulated with a range of TNF-α concentrations (0, 10, 25, 50 ng/mL) in FLSs. **(n,o)** Western blot analysis of nuclear IRF1 expression in TNF-α-treated (25 ng/mL) and untreated RA-FLSs. **(p)** Representative images of IRF1 immunofluorescence in FLSs treated with or without TNF-α (25 ng/mL). Bar = 10 μm. **(q)** Analysis of IRF1 immunofluorescence staining of **(p)** All experiments were performed at least three times independently. The results are expressed as mean values. Student’s t-test was used for two-group comparisons; for three or more groups, data were first checked for normality and homogeneity of variances. One-way ANOVA was applied when assumptions were met, with appropriate post hoc tests (Dunnett’s for comparisons against a common control, or Tukey’s for all pairwise comparisons); otherwise, Kruskal–Wallis test was used. The values are shown as the mean ± SEM. *p < 0.05, **p < 0.01, ***p < 0.001, ****p < 0.0001. ns, not significant. Values of p < 0.05 were considered significant.

Following cell-type annotation of single cells within rheumatoid arthritis (RA) synovial tissue via UMAP dimensionality reduction visualisation, the distribution characteristics of major cell populations were clearly delineated. These encompassed fibroblast subpopulations (CD142+, DKK3+ and PRG4+ fibroblasts) alongside immune cell types (macrophages, monocytes and T cells). Distinct boundaries and compact clustering among populations indicate reliable cell type identification and robust data resolution (Figure 1a). Height-adjustable gene bubble heatmaps further elucidate cell-type-specific expression profiles, thereby validating annotation reliability (Figure 1b). IRF1 expression in fibroblasts is significantly higher than in macrophages and T cells. Although the difference compared to monocytes is not statistically significant, the number of positive cells is markedly increased, indicating a high enrichment of IRF1 in fibroblasts (Figure 1c). Box plots illustrated differences in IRF1 expression across balanced fibroblast subtypes (CD142+ fibroblasts, PRG4+ fibroblasts, DKK3+ fibroblasts). To eliminate potential interference from unequal original sample sizes across subtypes (CD142+: 310 cells; PRG4+: 409 cells; DKK3+: 2,362 cells), a random subsampling strategy was employed to standardise cell numbers across all three subtypes to the minimum sample size (310 cells). Differential expression analysis was then conducted based on this adjusted dataset. Results revealed a distinct and consistent expression pattern of IRF1 across fibroblast subtypes: CD142+ Fibroblasts exhibited the highest IRF1 expression levels (median 2.90), followed by PRG4+ Fibroblasts (median 2.74), with DKK3+ Fibroblasts displaying the lowest expression (median 2.76). This established an overall expression trend of CD142^+^ > PRG4^+^ > DKK3^+^, which was consistently validated across four independent random subsampling sensitivity analyses (p range = 0.0077–0.0756, mean p = 0.0354; 3/4 runs significant). This trend was further independently corroborated in the CIA model and *in vitro* functional assays, supporting its overall robustness. This indicates that balanced sampling did not alter the inherent expression ranking of IRF1 across fibroblast subpopulations, further confirming the robustness and reliability of these expression differences (Figure 1d). In the GSE77298 dataset, RA patients showed numerous differentially expressed genes compared to healthy controls, with IRF1 significantly upregulated in RA (p < 0.01). This further supports its potential functional significance in disease states (Figures 1e,f).

To verify differences in IRF1 expression in synovium from osteoarthritis and rheumatoid arthritis, we first performed immunohistochemical staining. Compared to osteoarthritis synovium, the synovial tissue from rheumatoid arthritis patients exhibited significantly higher IRF1 expression (Figures 1g,h). Subsequently, we established a collagen-induced mouse arthritis model. Immunohistochemical analysis revealed that IRF1 expression was markedly elevated in the joints of collagen-treated mice relative to the normal control group (Figures 1i,j). Researchers successfully established a rheumatoid arthritis cell model by stimulating RA-FLSs with TNF-α. It was further confirmed by RT-qPCR, Western blot, and immunofluorescence staining that the expression of IRF1 in RA-FLSs treated with TNF-α was upregulated, consistent with research results in human samples and animal models (Figures 1k–o). IRF1, as a transcription factor, primarily exerts its physiological functions in the cell nucleus. Western blot analysis revealed that IRF1 levels in the nucleus were significantly elevated after exposure to TNF-α (Figures 1p,q). Taken together, these findings suggest that elevated IRF1 expression may contribute to the pathogenesis of rheumatoid arthritis.

### 3.2 Knockdown of IRF1 inhibits the pyroptosis of FLSs

**FIGURE 2:**
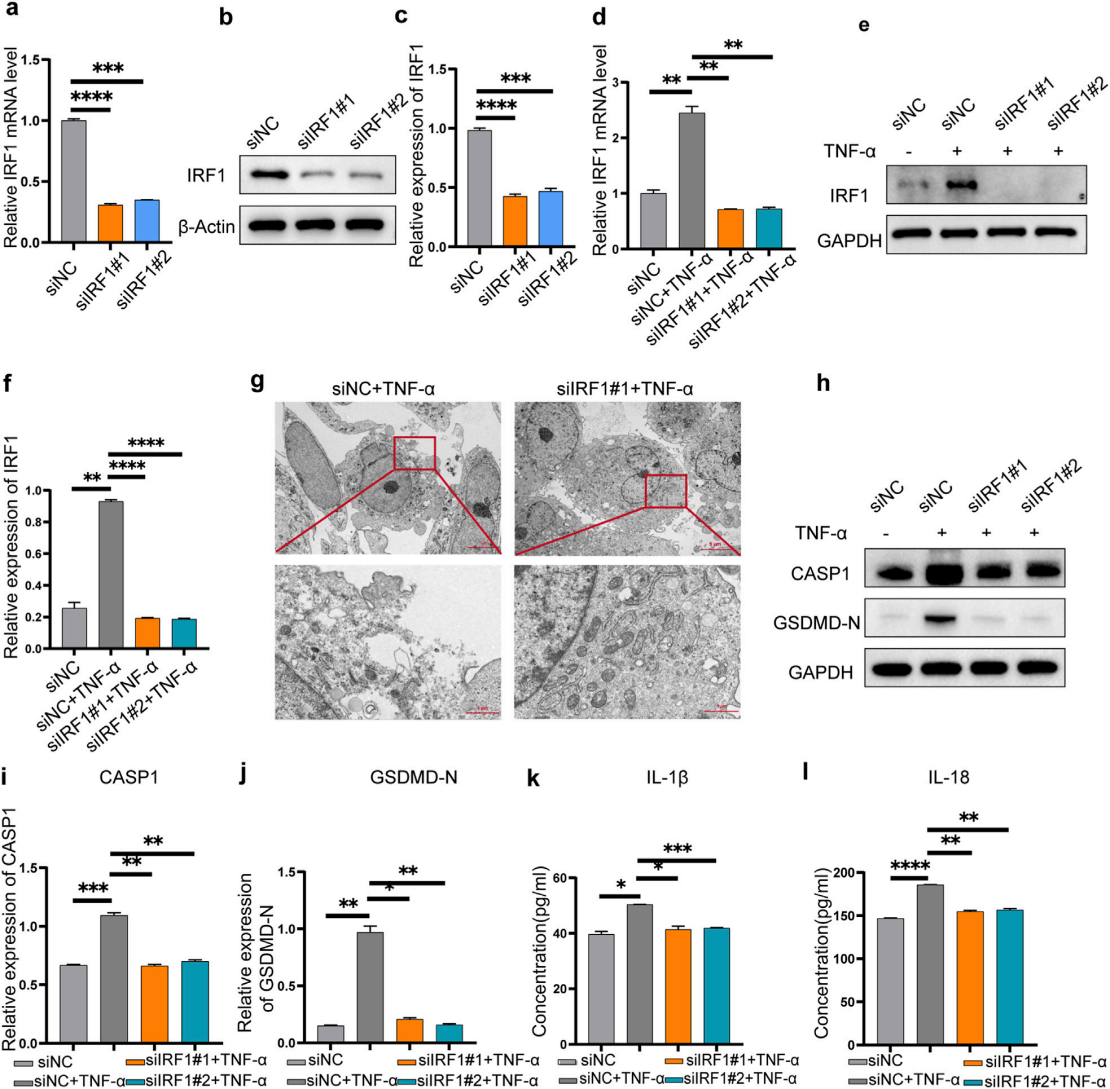
Inhibition of IRF1 alleviates pyroptosis in FLSs treated with TNF-α. **(a)** RT-qPCR analysis of relative IRF1 mRNA expression in FLSs transfected with IRF1 siRNA (siIRF1#1 and siIRF1#2) or negative control siRNA (siNC) **(b,c)** Western blot analysis of IRF1 protein expression in FLSs transfected with IRF1 siRNA or negative control siRNA. **(d)** RT-qPCR analysis of relative IRF1 mRNA expression in FLSs transfected with siIRF1 or siNC with or without TNF-α (25 ng/mL) stimulation **(e,f)** Western blot analysis of IRF1 protein expression in FLSs transfected with siIRF1 or siNC with or without TNF-α (25 ng/mL) stimulation. **(g)** Pyroptosis of TNF-α-treated FLSs transfected with siIRF1 and siNC was detected by transmission electron microscopy **(h–j)** Western blot analysis of CASP1 and GSDMD-N protein expression in FLSs transfected with siIRF1 or siNC with or without TNF-α (25 ng/mL) stimulation **(k,l)** ELISA analysis of IL-1β, IL-18 secretion in FLSs after IRF1 knockdown with or without TNF-α (25 ng/mL) stimulation. All experiments were performed at least three times independently. The results are expressed as mean values. Student’s t-test was used for two-group comparisons; for three or more groups, data were first checked for normality and homogeneity of variances. One-way ANOVA was applied when assumptions were met, with Dunnett’s for comparisons against a common control; otherwise, Kruskal–Wallis test was used. The values are shown as the mean ± SEM. *p < 0.05, **p < 0.01, ***p < 0.001. ns, not significant. Values of p < 0.05 were considered significant.

To investigate the effect of IRF1 on pyroptosis in RA-FLS, IRF1 knockdown was first achieved in RA-FLS via transfection with IRF1-specific siRNA, and its knockdown efficiency was evaluated (Figures 2a–c). Similarly, in TNF-α-treated FLS, IRF1 siRNA was added, and IRF1 could still be knocked down, as verified at the RNA and protein levels (Figures 2d–f). It was confirmed by transmission electron microscopy that knocking down IRF1 could significantly inhibit the pyroptosis of FLS. Treatment with TNF-α could promoted pyroptosis of FLS, while knockdown of IRF1 could inhibited pyroptosis induced by TNF-α (Figure 2g). Consistently, WB and ELISA results indicated that in TNF-α-treated FLS with IRF1 gene silencing, the expression levels of pyroptosis proteins CASP1, GSDMD-N, IL-1β, and IL-18 continued to decrease. Taken together, inhibiting IRF1 reduces pyroptosis in FLS (Figures 2h–l).

### 3.3 Local depletion of IRF1 alleviates joint inflammation and pyroptosis-related protein expression in the synovium of collagen-induced mice

**FIGURE 3:**
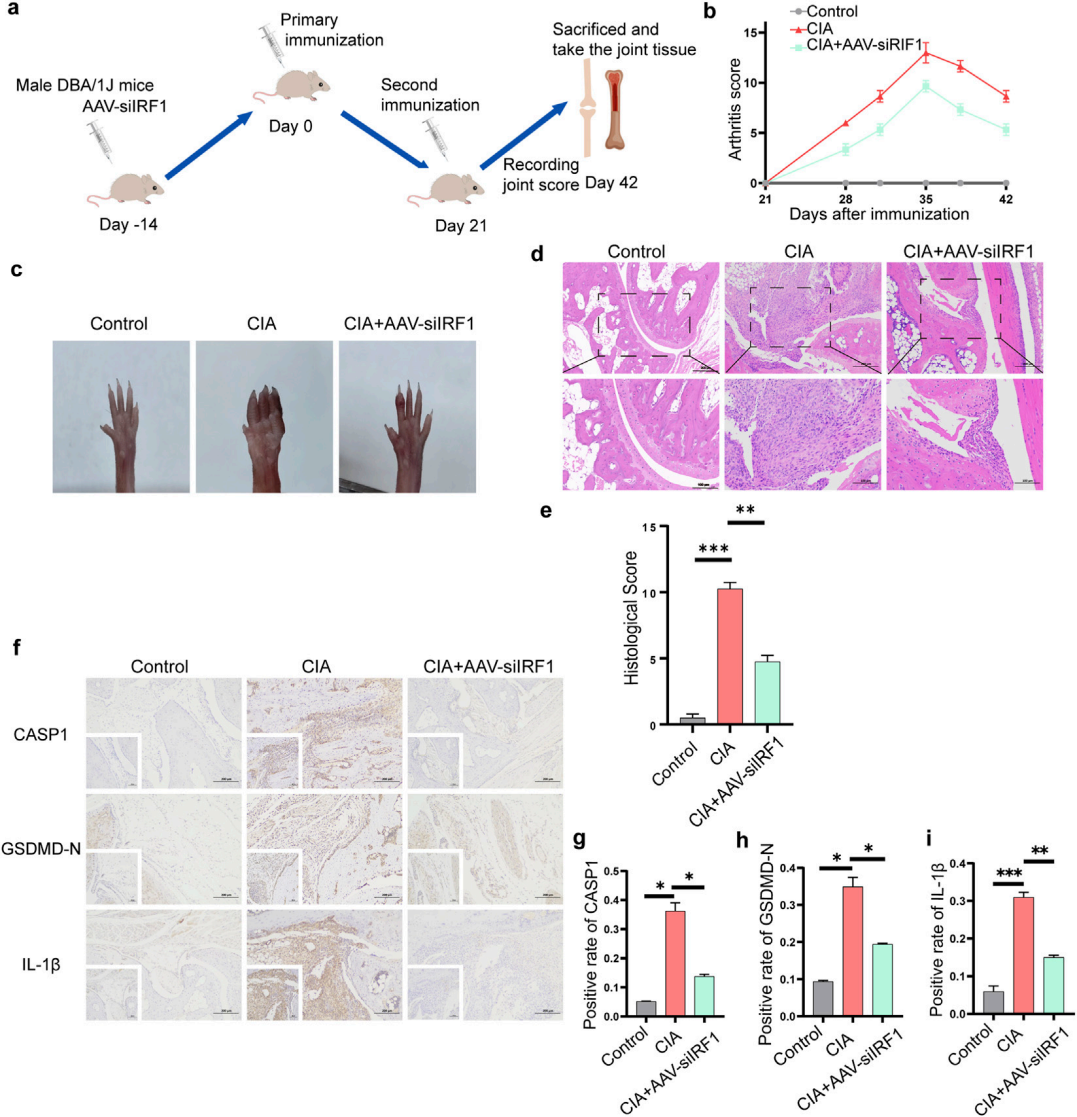
Local IRF1 knockdown attenuates collagen-induced arthritis. **(a)** Schematic diagram of the CIA mouse model and AAV-siIRF1 intervention treatment. **(b)** Arthritis scores in control, CIA model, and AAV-siIRF1-treated groups (n = 4 per group). **(c)** The photographs of the hind paw. **(d)** Representative H&E staining in each group (n = 4 per group). Bar = 200 μm (upper) or 100 μm (lower).**(e)** Histopathological analysis of H&E staining. Semiquantitative scores for inflammatory cell infiltration, synovial hyperplasia, and bone destruction were assessed and graded on a scale of 0 (normal) to 3 (severe) for four paws for the histological score. **(f)** Representative images of CASP1, GSDMD-N, and IL-1β staining by immunohistochemistry in the control group, CIA group, CIA + AAV-siIRF1 group (n = 4 per group). Bar = 100 μm (left) or 200 μm (right) **(g–i)** Analysis of CASP1, GSDMD-N, and IL-1β immunohistochemistry staining of **(e)**. All experiments were performed at least three times independently. The results are expressed as mean values. Student’s t-test was used for two-group comparisons; for three or more groups, data were first checked for normality and homogeneity of variances. One-way ANOVA was applied when assumptions were met, with Dunnett’s for comparisons against a common control; otherwise, Kruskal–Wallis test was used. The values are shown as the mean ± SEM. *p < 0.05, **p < 0.01, ***p < 0.001. ns, not significant. Values of p < 0.05 were considered significant.

To investigate whether IRF1 deficiency attenuates the progression of inflammatory arthritis, we employed a collagen-induced arthritis (CIA) model. Two weeks prior to CIA induction, AAV-siIRF1 was administered into the hind paw via intra-articular injection. The remaining procedures were carried out in accordance with the established CIA modeling protocol. (Figure 3a). While arthritis scores peaked on day 35 in the CIA model group, this elevation was significantly suppressed in mice receiving AAV-siIRF1 (Figure 3b). The experimental group displayed milder swelling in the joints and soles of the feet compared to the CIA group, as evidenced by hind paw images (Figure 3c). Histological analysis with HE staining demonstrated extensive articular cartilage degradation and widespread inflammatory cell infiltration in the CIA model group, along with considerable synovial hyperplasia, when compared with controls. The administration of AAV-siIRF1 significantly ameliorated key pathological features in the ankle joints, including articular cartilage degradation, inflammatory cell infiltration, and pannus development (Figure 3d), and reduced histological scores (Figure 3e). To assess the impact of AAV-siIRF1 on pyroptosis in the rheumatoid arthritis synovium, we performed immunohistochemical staining to examine the levels of pyroptosis-related proteins—specifically CASP1, GSDMD-N, and IL-1β—in synovial tissues from the control, CIA, and AAV-siIRF1-treated groups. The results revealed that expression of all three markers was significantly elevated in the CIA group compared to controls. Notably, administration of AAV-siIRF1 led to a marked reduction in CASP1, GSDMD-N, and IL-1β levels within the synovium (Figures 3f–i). In summary, experimental evidence confirmed that localized IRF1 inhibition markedly reduces joint inflammation. This finding underscores the promising therapeutic implications of targeting IRF1 in rheumatoid arthritis.

### 3.4 TNF-α promotes the expression of pyroptosis-related proteins via p65 in RA

**FIGURE 4:**
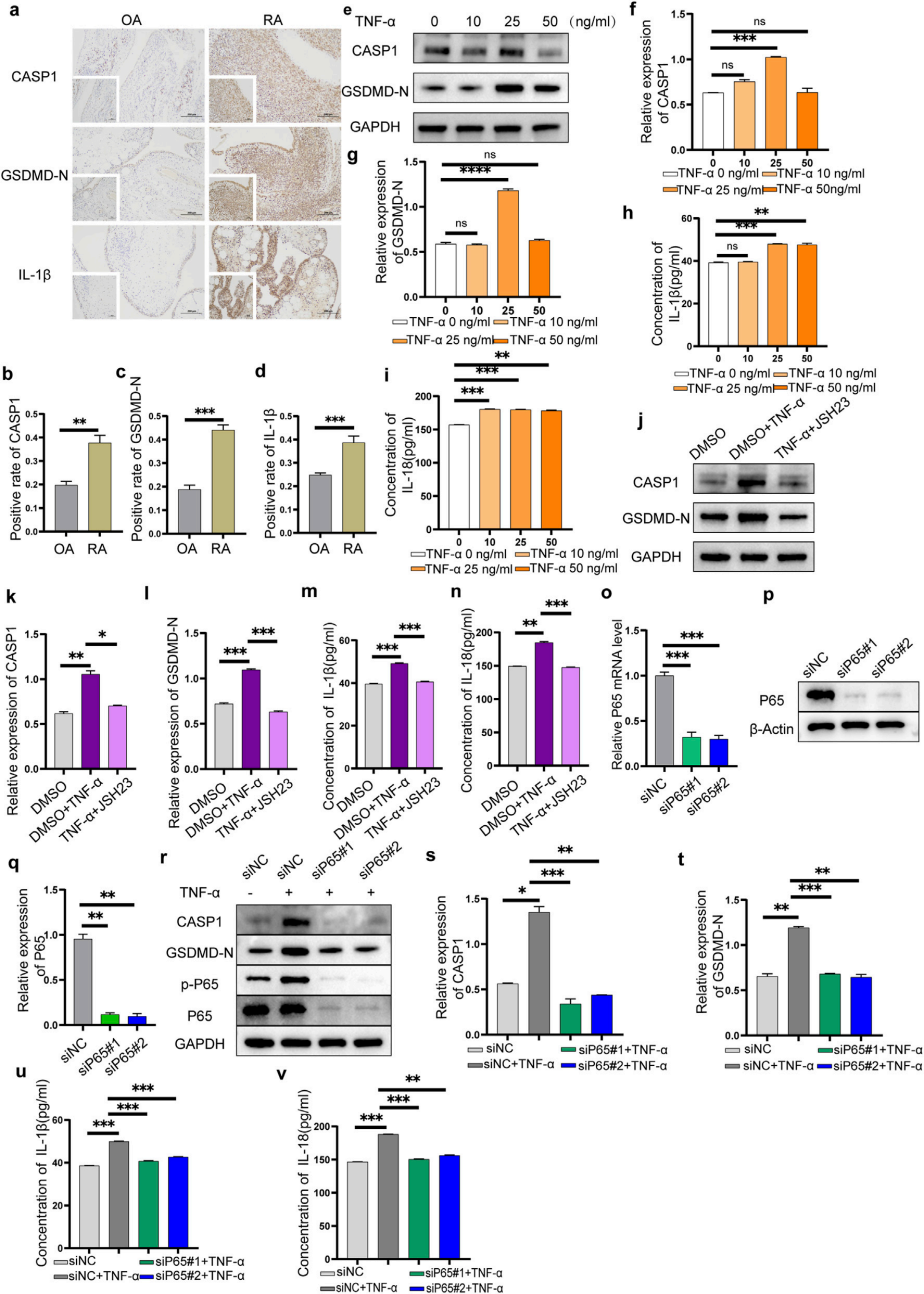
TNF-α activates p65 to promote pyroptosis of FLSs. **(a)** Representative images of CASP1,GSDMD-N, and IL-1β staining by immunohistochemistry in synovial tissue of Rheumatoid arthritis (RA, n = 8) and Osteoarthritis (OA, n = 5). Bar = 100 μm (left) or 200 μm (right). **(b–d)** Analysis of CASP1,GSDMD-N,IL-1β immunohistochemistry staining of (a). **(e–g)** Western blot analysis of CASP1 and GSDMD-N expression in fibroblast-like synoviocytes treated with increasing concentrations of TNF-α (0, 10, 25, and 50 ng/mL). **(h,i)** ELISA analysis of IL-1β and IL-18 secretion in fibroblast-like synoviocytes treated with TNF-α (0, 10, 25, 50 ng/mL). **(j–l)** Western blot analysis of CASP1,GSDMD-N protein expression in FLSs treated with DMSO, DMSO and TNF-α (25 ng/mL), TNF-α (25 ng/mL), and JSH23 separately. **(m,n)** ELISA analysis of IL-1β, IL-18 secretion treated with DMSO, DMSO and TNF-α (25 ng/mL), TNF-α (25 ng/mL) and JSH23 in FLSs separately. **(o)** RT-qPCR analysis of relative p65 mRNA expression in FLSs transfected with p65 siRNA (sip65#1 and sip65#2) or negative control siRNA (siNC). **(p,q)** Western blot analysis of p65 protein in FLSs after transfection with p65 siRNA or negative control siRNA. **(r–t)** Western blot analysis of CASP1 and GSDMD-N protein expression in FLSs transfected with sip65 or siNC with or without TNF-α (25 ng/mL) stimulation. **(u,v)** ELISA analysis of IL-1β, IL-18 secretion in FLSs after p65 knockdown with or without TNF-α (25 ng/mL) stimulation. All experiments were performed at least three times independently. The results are expressed as mean values. Student’s t-test was used for two-group comparisons; for three or more groups, data were first checked for normality and homogeneity of variances. One-way ANOVA was applied when assumptions were met, with Dunnett’s for comparisons against a common control; otherwise, Kruskal–Wallis test was used. The values are shown as the mean ± SEM. *p < 0.05, **p < 0.01, ***p < 0.001. ns, not significant. Values of p < 0.05 were considered significant.

To investigate the expression of pyroptosis-related proteins in rheumatoid arthritis, we initially performed immunohistochemical staining on synovial tissues obtained from OA and RA patients. Comparative analysis revealed that the synovial tissues from RA patients exhibited markedly upregulated levels of pyroptosis-associated markers, including CASP1, GSDMD-N, and IL-1β, relative to those from OA patients (Figures 4a–d). Consistent with results from human specimens, Western blot and ELISA assays revealed that pyroptosis-associated protein expression in RA-FLSs increased with rising TNF-α concentrations, peaking at 25 ng/mL (Figures 4e–i). We observed that administration of JSH23, a selective NF-κB inhibitor, effectively suppressed the production of key pyroptosis-related proteins—including CASP1, GSDMD-N, IL-1β, and IL-18—in RA-FLSs challenged with TNF-α, relative to untreated cells (Figures 4j–n). A gene-silencing approach was used to investigate the role of p65 in pyroptosis in RA-FLSs. This involved transfecting the cells with siRNA targeting p65 and subsequently validating knockdown efficiency (Figures 4o–q). Western blotting and ELISA analyses consistently revealed that knockdown of p65 resulted in a sustained reduction in the levels of CASP1, GSDMD-N, IL-1β, and IL-18 in TNF-α-treated RA-FLSs (Figures 4r–v). Collectively, these findings demonstrate that TNF-α facilitates pyroptosis in RA-FLS through the p65 pathway.

### 3.5 TNF-α regulates IRF1 expression via p65 in FLS

**FIGURE 5:**
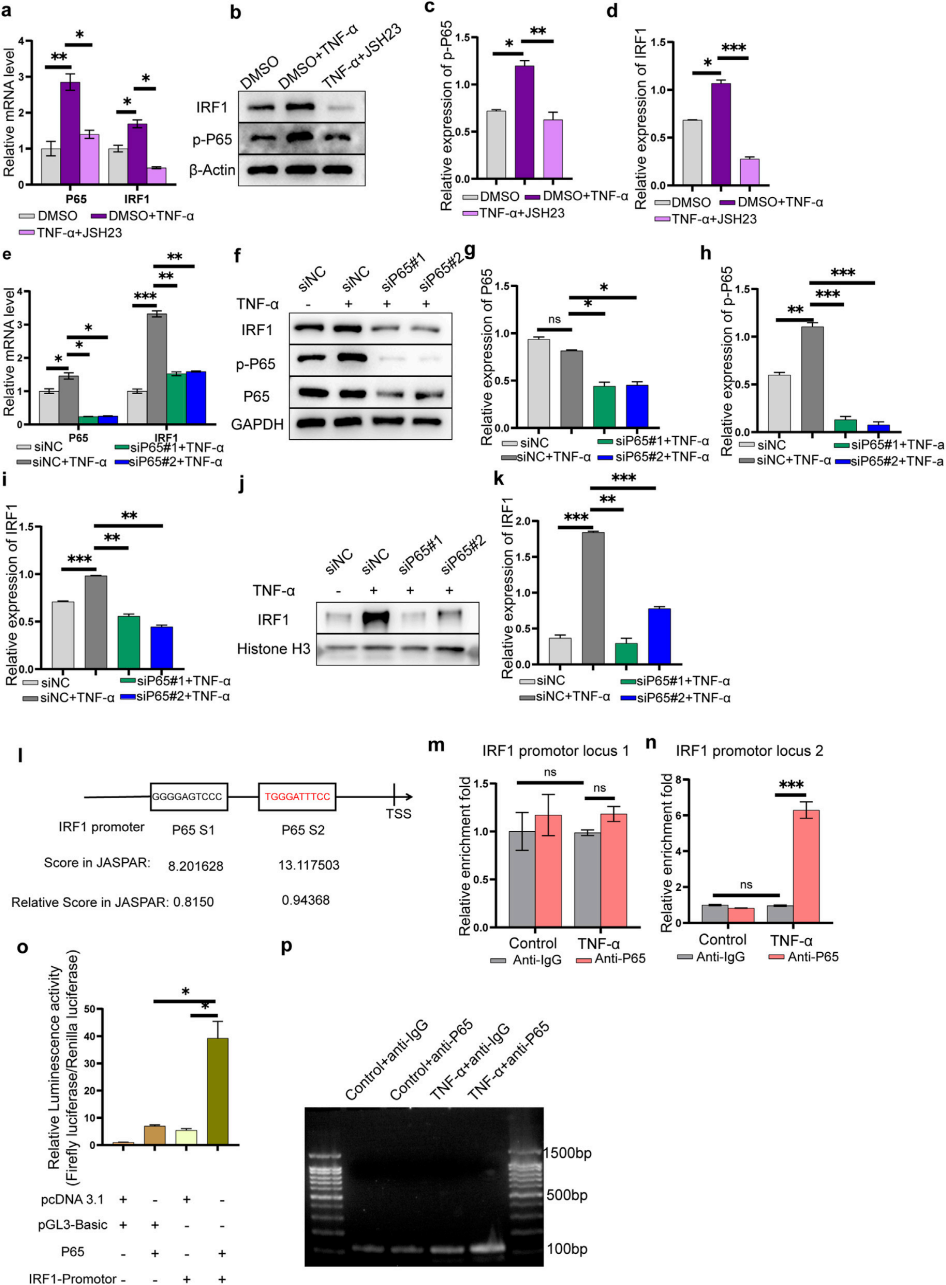
TNF-α promotes IRF1 expression via p65. **(a)** RT-qPCR analysis of relative p65 and IRF1 mRNA expression in FLSs treated with DMSO, DMSO and TNF-α (25 ng/mL), TNF-α (25 ng/mL), and JSH23 separately. **(b–d)** Western blot analysis of p-p65,IRF1 protein expression in FLSs treated with DMSO, DMSO and TNF-α (25 ng/mL), TNF-α (25 ng/mL), and JSH23 separately. **(e)** RT-qPCR analysis of relative p65 and IRF1 mRNA expression in FLSs transfected with sip65 or siNC with or without TNF-α (25 ng/mL) stimulation. **(f–i)** Western blot analysis of p65, p-p65, and IRF1 protein expression in FLSs transfected with sip65 or siNC with or without TNF-α (25 ng/mL) stimulation. **(j,k)** Western blot analysis of IRF1 protein expression in the nucleus, transfected with sip65 or siNC with or without TNF-α (25 ng/mL). (l) The JASPAR database was employed to predict p65 binding sites in the IRF1 promoter region; the top-ranked sites (S1 and S2) were subsequently chosen. **(m)** The CUT&Tag-qPCR experiment was applied to verify the enrichment efficiency of p65 at the predicted binding site one of the IRF1 promoter region (IRF promoter site 1). **(n)** The CUT&Tag-qPCR experiment was applied to verify the enrichment efficiency of p65 at the predicted binding site 2 of the IRF1 promoter region (IRF promoter site 2). **(o)** p65 transcriptional activity was assessed using a dual-luciferase reporter assay. **(p)** Fluorescence imaging of DNA enriched with p65 antibody was detected by DNA agarose gel electrophoresis experiment. All experiments were performed at least three times independently. The results are expressed as mean values. Student’s t-test was used for two-group comparisons; for three or more groups, data were first checked for normality and homogeneity of variances. One-way ANOVA was applied when assumptions were met, with Dunnett’s for comparisons against a common control; otherwise, Kruskal–Wallis test was used. The values are shown as the mean ± SEM. *p < 0.05, **p < 0.01, ***p < 0.001. ns, not significant. Values of p < 0.05 were considered significant.

Upon TNF-α treatment, RA-FLS displayed increased expression of p65 (RelA), p-p65, and IRF1 compared to untreated cells, consistent with prior observations. Following the addition of the NF-κB inhibitor JSH23, PCR and WB assays were employed to detect p65, p-p65, and IRF1 expression. It was observed that p65, p-p65 and IRF1 expression decreased synchronously, suggesting that IRF1 expression may be correlated with p65 (Figures 5a–d). To interrogate the putative upstream regulation of IRF1 by p65, p65 was silenced in TNF-α-induced FLS, resulting in reduced IRF1 expression levels as determined by PCR and Western blot assays (Figures 5e–i). Additionally, nuclear IRF1 expression showed the same trend: following p65 inhibition, IRF1 expression decreased (Figures 5j,k). Previous studies have demonstrated that p65 binds to the IRF1 promoter within scar tissue, thereby inducing IRF1 transcription (Gu et al., 2023). We next selected the two highest-priority candidate sites within the IRF1 promoter for primer design, following the identification of potential p65 binding regions using JASPAR software (Figure 5l). This approach, followed by CUT&Tag-qPCR analysis, confirmed that TNF-α stimulation significantly increased p65 occupancy at site 2 (Figures 5m,n). Subsequently, employing a dual-luciferase reporter system, we assessed the direct impact of p65 on the IRF1 promoter, which showed a marked increase in transcriptional activity upon p65 overexpression (Figure 5o). DNA agarose gel electrophoresis imaging revealed clear separation of loaded DNA fragments post-electrophoresis. The TNF-α+anti-p65 group exhibited markedly stronger fluorescence intensity than the other groups, consistent with both CUT&Tag-qPCR and dual luciferase reporter assay findings (Figure 5p). Collectively, these results suggest that IRF1 functions as a direct transcriptional target of p65.

### 3.6 IRF1 directly interacts with the CASP1 promoter and activates its transcription

**FIGURE 6:**
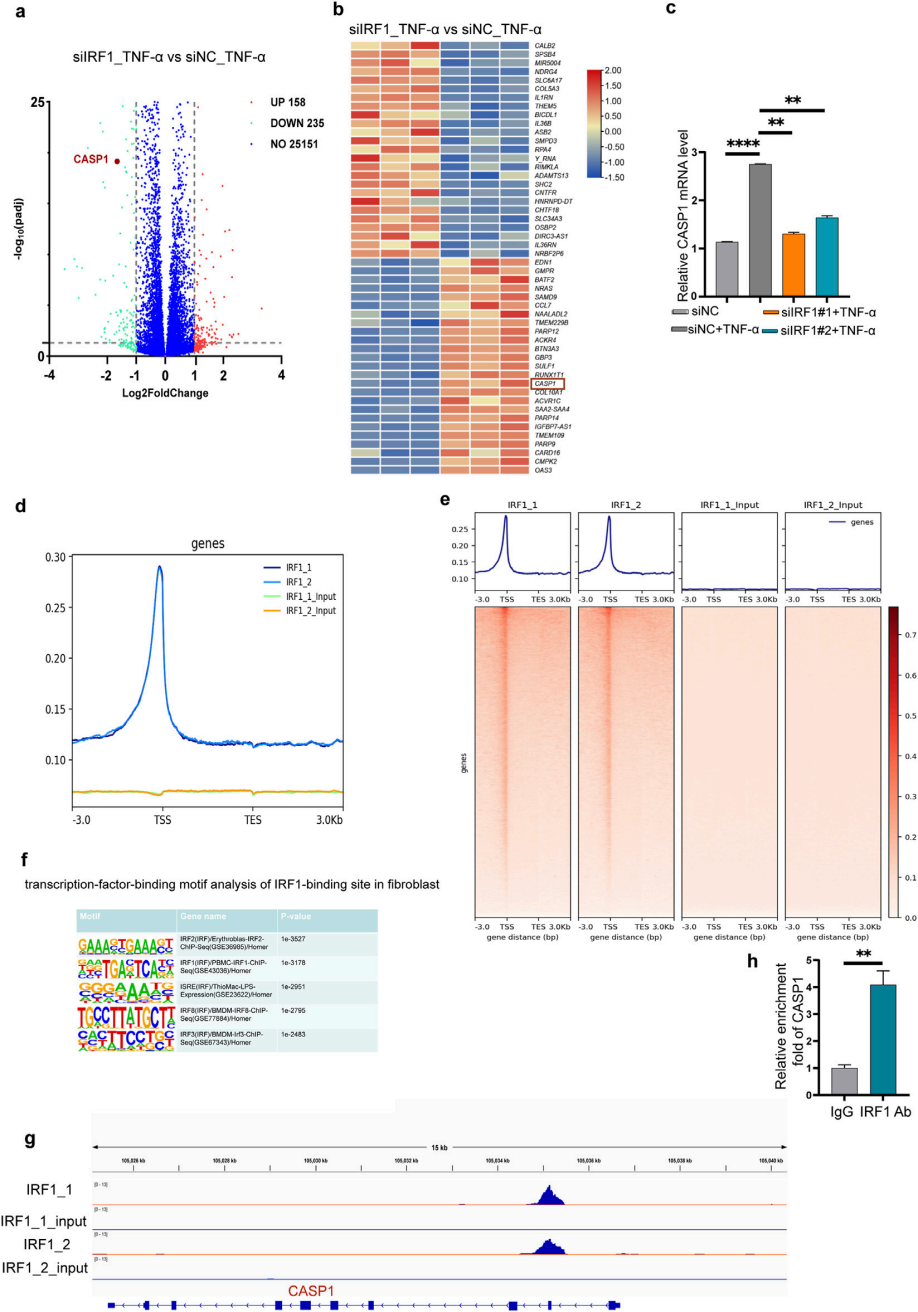
IRF1 directly targets CASP1. **(a)** Volcano plot for RNA sequencing data (P-adj <0.05, |log2FoldChange| > 1). When TNF-α-treated FLSs were transfected with siNC or siIRF1. **(b)** The heat map of the representative DEGs when TNF-α-treated FLSs were transfected with siNC or siIRF1. **(c)** Identification of differential gene CASP1 mRNA. **(d)** The distribution trend chart of ChIP-seq signals near TSS. **(e)** Chip-seq signal enrichment map near TSS. **(f)** The top five transcription factor binding sequences of IRF1. **(g)** IGV visualization of the regulatory regions of the IRF1_1 input (blue line) and IRF1_2 input (blue line), and the IRF1_1 (orange line) and IRF1_2 (orange line) specific signals at the indicated loci. **(h)** ChIP-qPCR analysis of predicted IRF1 binding sites in the CASP1 promoter. All experiments were performed at least three times independently. The results are expressed as mean values. Student’s t-test was used for two-group comparisons; for three or more groups, data were first checked for normality and homogeneity of variances. One-way ANOVA was applied when assumptions were met, with Dunnett’s for comparisons against a common control; otherwise, Kruskal–Wallis test was used. The values are shown as the mean ± SEM. *p < 0.05, **p < 0.01, ***p < 0.001. ns, not significant. Values of p < 0.05 were considered significant.

Subsequent bulk RNA-seq analysis confirmed the upstream-downstream regulatory relationship between IRF1 and CASP1. In IRF1-knockdown RA-FLS, a total of 393 differentially expressed genes (DEGs) were identified relative to controls, comprising 235 downregulated and 158 upregulated genes (Figure 6a). The heat map shows representative DEGs with absolute fold changes (Figure 6b). Consistent with the sequencing data, RT-qPCR analysis confirmed that CASP1 mRNA levels were reduced upon IRF1 knockdown in TNF-α-treated FLS (Figure 6c). To investigate the mechanism underlying IRF1-mediated CASP1 upregulation, we performed ChIP-seq to uncover direct transcriptional targets of IRF1. This approach involved immunoprecipitating IRF1-bound DNA fragments and subjecting the enriched samples to high-throughput sequencing. By standardising the IRF1-Ab group against the IgG control group, it was found that the specific peaks in both IRF1-Ab groups were predominantly located within the promoter region (Figures 6d,e). The binding of regulatory proteins, such as transcription factors, to DNA requires recognition of specific motifs to exert their regulatory functions. Analysis of Peak-associated sequences using Homer software yields enriched motifs and their corresponding transcription factors. The figure depicts the top five motifs with the smallest p-values and their associated genes (Figure 6f). IGV screenshots reveal the regulatory regions of the two specimens, IRF1_1 and IRF1_2, along with signals enriched in the CASP1 promoter region (Figure 6g). We also performed Cut-Run validation on CASP1, further confirming that the IRF1 protein binds its promoter (Figure 6h). Based on the above evidence, IRF1 regulates CASP1 transcription via direct promoter binding.

## 4 Discussion

This study demonstrates that IRF1 expression is markedly elevated in the synovium of rheumatoid arthritis joints. TNF-α increases IRF1 expression via the NFκB pathway, thereby promoting pyroptosis. Experiments demonstrated that local inhibition of IRF1 effectively alleviates joint inflammation in collagen-induced mice. IRF1 directly binds the CASP1 promoter to enhance transcription. The present results highlight the essential function of the TNF-α/IRF1/CASP1 signaling axis in driving rheumatoid arthritis pathogenesis. A study (Su et al., 2025) identified CD142+ fibroblasts as key effector cells in meniscal damage of knees affected by RA. Enriched within the RA synovium and migrating from the sublining to the lining layer. They mediate meniscal structural destruction through highly invasive and proliferative mechanisms, and their high proportion within the synovial lining layer constitutes a risk factor for RA patients ultimately requiring total knee arthroplasty.

Our study reveals the significant importance of IRF1 being highly expressed in CD142^+^ synovial fibroblasts (with expression levels markedly higher than other fibroblast subtypes such as PRG4^+^ and DKK3^+^). As a classic inflammation-associated transcription factor, the elevated expression of IRF1 in CD142^+^ synovial fibroblasts not only suggests that this transcription factor may enhance its role in mediating meniscal damage by regulating inflammatory activation and invasion-related gene expression in CD142^+^ cells, but also provides key molecular clues for elucidating the activation regulatory mechanisms of CD142^+^ synovial fibroblasts.

Accumulating evidence has established a significant regulatory function for IRF1 in the pathogenesis of various inflammatory disorders. Research indicates that ionising radiation activates IRF1 via the mtDNA-cGAS/STING signalling pathway. Acting as a transcription factor, IRF1 enhances CASP1 transcription, thereby exacerbating inflammatory damage (Geng et al., 2024a). In osteoarthritis, activated IRF1 directly binds to the PARP12 promoter, leading to its upregulation. This elevated expression of PARP12 (poly (ADP-ribose) polymerase family member 12) subsequently inhibits mitochondrial autophagy, thereby promoting cartilage degradation (Deng et al., 2024). The IFN-γ/IRF1-KPNA4 (Karyopherin Alpha 4, KPNA4) pathway induces elevated CXCL10 (C-X-C motif chemokine ligand 10, CXCL10) levels, driving interactions between human umbilical cord mesenchymal stem cells (hUC-MSCs) and the lupus nephritis immune microenvironment (Zhang et al., 2024b). These studies collectively demonstrate that IRF1 expression increases in inflammatory diseases, typically functioning as a transcription factor. Consistent with our findings, IRF1 is upregulated in RA and functions as a transcription factor that enhances CASP1 transcriptional activity, consequently promoting pyroptosis in RA-FLSs.

We propose that suppressing IRF1 attenuates pyroptosis in RA-FLSs. Our data indicate that IRF1 knockdown mitigates TNF-α-induced pyroptosis, correlating with reduced expression of CASP1, GSDMD-N, IL-1β, and IL-18. Mechanistically, CASP1 acts as an early trigger of pyroptosis: upon activation, it cleaves GSDMD to generate GSDMD-N. Multiple GSDMD-N molecules then oligomerize to form pore-forming complexes that insert into the cell membrane, enabling the release of mature IL-1β and IL-18 into the extracellular space (Bai et al., 2025). Our study establishes a causal link between IRF1 expression and pyroptosis in RA-FLSs, thereby identifying a potential therapeutic target for understanding the mechanisms underlying pyroptosis in these cells.

We further observed that TNF-α induces pyroptosis in RA-FLSs through p65 activation, with phosphorylated p65 functioning as a transcription factor to upregulate IRF1 expression. The NF-κB pathway, in which p65 is a key component, has been extensively implicated in the pathogenesis of rheumatoid arthritis (Loh et al., 2019; Lee et al., 2020). Recent studies have also shown that the NF-κB pathway is tightly linked to pyroptosis and positively regulates its initiation in various disease contexts. H3K18 lactylation-mediated nucleotide-binding oligodomain-2 (NOD-2) induces astrocytic pyroptosis via the NF-κB pathway, thereby promoting inflammation (Li et al., 2025). NBD peptides inhibit ASMase-mediated LMP and autophagy suppression via NF-κB and its downstream cascade amplification, thereby suppressing neuronal pyroptosis after spinal cord injury and aiding functional recovery (Geng et al., 2024b). This aligns with our findings. Our findings indicate that TNF-α activates the NF-κB inflammatory pathway, thereby promoting the expression of pyroptosis-associated proteins in RA-FLSs. This conclusion supports the notion that the NF-κB pathway promotes pyroptosis. More innovatively, our study links NF-κB and IRF1 through pyroptosis and elucidates their interaction mechanism: p65 directly acts on the IRF1 promoter, upregulating IRF1 expression by promoting transcription and enhancing downstream CASP1-mediated pyroptosis. Our study provides fresh insights into the NF-κB–IRF1 axis in pyroptosis, while also revealing that IRF1-dependent pyroptosis varies in a tissue- and disease-specific manner.

Importantly, we have identified CASP1 as a critical downstream effector of IRF1 in rheumatoid arthritis. As a transcription factor, IRF1 binds directly to the CASP1 promoter, driving its transcriptional expression and consequently amplifying pyroptosis in RA-FLSs.The synchronous upregulation of IRF1 and CASP1 in intestinal epithelial cell pyroptosis during Crohn’s disease suggests that these two molecules are key pyroptosis-associated biomarkers. (Xu et al., 2025). Deficiency in ATP-binding cassette transporter A1 (ABCA1) reduces foot cell pyroptosis initiation in diabetic kidney disease (DKD) by inhibiting the IRF1/CASP1 axis (Ito et al., 2023). Nur77 suppresses IRF1 expression and its subsequent binding to the CASP1 promoter, thereby inhibiting pyroptosis in pulmonary vascular endothelial cells (Ding et al., 2021). A recent study reveals that IRF1, induced by Th1 cells in autoimmune myocarditis, directly engages the CASP1 promoter to drive its transcription. This event promotes pyroptosis, resulting in cardiac damage and impaired function (Zhang et al., 2025b). These studies, all of which rely on the IRF1/CASP1 pathway to regulate pyroptosis, provide theoretical support for our research. More significantly, we elucidated that the upstream TNF-α/NF-κB pathway and downstream CASP1-activated pyroptosis are linked via an IRF1-dependent pathway, suggesting that IRF1 inhibitors may represent a potential therapeutic target for rheumatoid arthritis.

Beyond FLSs, pyroptosis in RA extends to other key cell types, particularly macrophages, which are abundant in the inflamed synovium and play a critical role in inflammatory amplification. Pyroptosis in RA extends well beyond FLSs. Macrophages—among the most abundant and functionally active innate immune cells in the inflamed synovium—are equally prone to pyroptotic death, and their demise carries substantial consequences for inflammatory amplification and joint destruction. Unlike FLSs pyroptosis, which relies on diverse upstream signals, macrophage pyroptosis in RA is predominantly driven by NLRP3 inflammasome activation, with IL-1β and IL-18 maturation serving as its principal downstream outputs. IRF1 has emerged as a critical upstream orchestrator of this process. In macrophages, IRF1 engages the ROS-dependent NLRP3–ASC axis to drive caspase-1 activation, GSDMD-N cleavage, and the maturation of IL-1β and IL-18; accordingly, IRF1 overexpression amplifies pyroptotic signaling, while its inhibition blunts inflammatory cell death (Guo et al., 2019). Disease-specific evidence reinforces this picture: in autoimmune myocarditis, IRF1 was found to bind directly to the CASP1 promoter, boosting caspase-1 transcription in M1 macrophages and triggering pyroptosis (Zhang et al., 2025b); in atherosclerosis, IRF1 occupies the promoter regions of both GSDMD and CASP1, coordinately elevating their expression to fuel NLRP3 inflammasome-driven pyroptotic cell death (Fan et al., 2023). Taken together, these observations point to IRF1-mediated macrophage pyroptosis as a regulatory logic conserved across immunologically distinct disease contexts. Beyond IRF1, two additional layers of control over macrophage pyroptosis in RA have recently come into focus. ANGPTL2 has been identified as an indispensable guardian of macrophage mitophagy: its deficiency disrupts IGFBP5-dependent mitophagic flux, allows dysfunctional mitochondria to accumulate, and ultimately sustains NLRP3 inflammasome activation—a cascade that can be reversed by intra-articular AAV-Angptl2 delivery, which restores mitophagy, suppresses pyroptosis, and ameliorates arthritis (Liu et al., 2026). Separately, DNA polymerase β (Pol β), a core enzyme in base excision repair, is markedly reduced in peripheral blood mononuclear cells from active RA patients and CIA mice. Loss of Pol β allows DNA damage to accumulate and cytosolic dsDNA to leak, activating the cGAS–STING–NF-κB axis and upregulating NLRP3, IL-1β, and IL-18 to drive macrophage pyroptosis (Gu et al., 2022). Whether IRF1 directly governs macrophage pyroptosis in RA through mechanisms analogous to those characterized in FLSs remains an open question. Equally unexplored is the pyroptosis-mediated crosstalk between FLSs and macrophages—a dialogue that, given each cell type’s capacity for pyroptotic amplification, may be a pivotal mechanism sustaining chronic synovial inflammation and merits dedicated investigation.

Several limitations of this study should be considered when interpreting our findings. First, the sample size of human synovial tissues used for immunohistochemistry and qPCR validation in this study is relatively limited (RA n = 8, OA n = 5), which may affect statistical power. Although our results are consistent with multiple independent single-cell transcriptomic datasets and have been cross-validated in the CIA model and *in vitro* experiments, future independent validation in larger RA patient cohorts is warranted to confirm the clinical relevance of this signaling axis and to establish its potential as a therapeutic target. In addition to the sample size limitation, the choice of the animal model also warrants discussion. It should be acknowledged that although the collagen-induced arthritis model is one of the most widely used preclinical models for RA, it does not perfectly recapitulate all aspects of human RA, particularly the chronic, relapsing-remitting disease course (Brand et al., 2007). The CIA model exhibits acute, self-limiting polyarthritis, which contrasts with the chronic nature of human RA. Moreover, it shows considerable variability in incidence, severity, and inter-group consistency; it can only be induced in susceptible rodent strains, with low incidence and high variability in C57BL/6 mice; and the higher incidence in males, as opposed to the female predominance in human RA, represents another notable discrepancy (Damerau and Gaber, 2020). In addition, the CIA model is predominantly driven by Th1/Th17-mediated autoimmune responses, whereas human RA exhibits a more heterogeneous immunopathological profile. Therefore, our findings warrant further validation in other models that more closely mimic human RA, such as the K/BxN serum-transfer model, humanized mouse models, or transgenic models. Nevertheless, the CIA model still provides a robust proof-of-concept at the current stage for the therapeutic strategy of targeting the IRF1-mediated FLSs pyroptosis pathway. Beyond the translational constraints of the CIA model, we also acknowledge a technical limitation concerning the mechanistic characterization of pyroptosis. The pyroptosis-associated phenotypes reported in this study should not be equated with definitive confirmation of the classical CASP1/GSDMD-dependent pyroptotic pathway. We acknowledge that cleaved-CASP1 and the N-terminal fragment of GSDMD (GSDMD-N) are critical direct hallmarks of pyroptosis execution, and their stable detection constitutes an indispensable step in subsequent validation efforts. In the current phase of this study, due to antibody specificity and technical constraints, reproducible and statistically reliable results for cleaved caspase-1 could not be obtained across multiple independent replicates. Furthermore, the lytic cell death features induced by TNF-α stimulation in RA-FLSs cannot completely exclude the possible overlapping involvement of other lytic cell death pathways, such as necroptosis or ferroptosis. Therefore, the conclusions of this study should be regarded as preliminary findings; our results suggest that IRF1 promotes pyroptosis-associated inflammatory responses in RA-FLSs, but do not constitute conclusive proof of the classical pyroptotic pathway. Future studies incorporating stable detection of cleaved proteins, gene knockout approaches, and specific inhibitor interventions are warranted to further establish the integrity and exclusivity of this pathway. In summary, this study reveals the pivotal role of the TNF-α-IRF1-CASP1 axis in the pathogenesis of rheumatoid arthritis and provides novel insights for targeting IRF1 as a therapeutic intervention.

## Data Availability

The original contributions presented in the study are included in the article/supplementary material, further inquiries can be directed to the corresponding authors.
